# The Physiological Roles of Amyloid-β Peptide Hint at New Ways to Treat Alzheimer's Disease

**DOI:** 10.3389/fnagi.2018.00118

**Published:** 2018-04-25

**Authors:** Holly M. Brothers, Maya L. Gosztyla, Stephen R. Robinson

**Affiliations:** ^1^Department of Psychology, The Ohio State University Columbus, Columbus, OH, United States; ^2^Department of Neuroscience, The Ohio State University Columbus, Columbus, OH, United States; ^3^Discipline of Psychology, School of Health and Biomedical Sciences, RMIT University, Melbourne, VIC, Australia

**Keywords:** infection, antimicrobial, cancer, traumatic injury, cerebrovascular, immune system, seizure, ARIA

## Abstract

Amyloid-ß (Aß) is best known as the misfolded peptide that is involved in the pathogenesis of Alzheimer's disease (AD), and it is currently the primary therapeutic target in attempts to arrest the course of this disease. This notoriety has overshadowed evidence that Aß serves several important physiological functions. Aß is present throughout the lifespan, it has been found in all vertebrates examined thus far, and its molecular sequence shows a high degree of conservation. These features are typical of a factor that contributes significantly to biological fitness, and this suggestion has been supported by evidence of functions that are beneficial for the brain. The putative roles of Aß include protecting the body from infections, repairing leaks in the blood-brain barrier, promoting recovery from injury, and regulating synaptic function. Evidence for these beneficial roles comes from *in vitro* and *in vivo* studies, which have shown that the cellular production of Aß rapidly increases in response to a physiological challenge and often diminishes upon recovery. These roles are further supported by the adverse outcomes of clinical trials that have attempted to deplete Aß in order to treat AD. We suggest that anti-Aß therapies will produce fewer adverse effects if the known triggers of Aß deposition (e.g., pathogens, hypertension, and diabetes) are addressed first.

## Introduction

The presence of large numbers of “senile plaques” in the hippocampus and overlying cortical regions is one of the definitive features of Alzheimer's disease (AD). These spherical proteinaceous deposits consist primarily of a 38–42 amino acid long peptide known as amyloid-ß (Aß). The Aß peptide is derived from a transmembrane protein called amyloid-β precursor protein (APP). β-site APP cleaving enzyme 1 (BACE1) cleaves APP to release the C99 fragment of APP. This fragment gives rise to various species of Aβ peptide during subsequent cleavage by γ-secretase. In the brain, Aß is produced by astrocytes and neurons; however, non-neural tissues such as skin, skeletal muscle and intestinal epithelium also secrete Aß (Puig and Combs, 2013). Normally present in a soluble form, Aß is secreted into the extracellular space of the brain and then cleared by the cerebrospinal fluid (CSF) and vascular system. In the CSF of cognitively normal humans, the most abundant isoform is 40 amino acids long (Aß_40_; 2–3 ng/mL), while the second most common isoform (Aß_42_) is present at approximately 0.75 ng/mL (Ida et al., 1996; Mo et al., 2015). Experiments with transgenic mice that overexpress Aß have revealed that the turnover of soluble Aß is rapid, and it is cleared from the extracellular space and CSF with a half-life of just 0.7–2.0 h (Savage et al., 1998; Abramowski et al., 2008). Radioactive tracer studies have shown that Aß is removed from the circulation by the capillary beds of the kidneys, liver, gastrointestinal tract, and skin (Xiang et al., 2015).

Soluble Aß can bind to other molecules of Aß to form oligomers that are cleared more slowly from the brain, or which can accrete to form insoluble Aß plaques. Numerous *in vivo* and *in vitro* experiments demonstrated that the oligomeric and insoluble forms of Aß are toxic to brain cells. These findings have led to the prevailing view that Aß exhibits a “toxic gain-of-function” when it forms oligomers and aggregates into plaques, thereby directly contributing to the pathogenesis of AD, and making it the logical target for therapeutic intervention (Masters and Selkoe, 2012). However, of more than 200 clinical trials that specifically targeted Aß between 1984 and 2013, none improved clinical outcomes in AD patients (Schneider et al., 2014). Indeed, some of these trials were associated with adverse outcomes. This situation has continued through to the present day, with not a single clinical trial between 2012 and 2017 producing a significant cognitive benefit. This frustrating lack of progress has led to suggestions that Aß needs to be targeted at an earlier stage of the disease, prior to the onset of dementia or even before any cognitive changes are detectable (Tarawneh and Holtzman, 2009).

The term “amyloidogenic” is applied to any soluble peptide or protein that has the capacity to interact with similar molecules to self-assemble into insoluble fibrils, which then bond with other fibrils to form a regular β-pleated sheet. The molecular conformation of these amyloid sheets makes them strongly resistant to degradation by proteolytic enzymes. Functional amyloids and amyloidogenic peptides are common in biological systems. For instance, colonial bacteria utilize amyloids to aggregate, attach to a substrate, and improve the strength of their protective biofilms (Dueholm et al., 2013). Plants produce amyloids with strong antifungal and antimicrobial properties (Villar-Piqué et al., 2010; Garvey et al., 2013).

A meta-analysis of APP-like and Aß-like sequences in living species has found that these sequences are present in hydra and sea anemones, indicating that the sequences must have evolved prior to the evolution of arthropods, around 500 million years ago (Tharp and Sarkar, 2013). All vertebrates produce APP, ß-secretase, and Aß; Aß in birds, reptiles and amphibians has a >90% sequence homology with human Aß, while in mammals the sequence homology exceeds 95% (Tharp and Sarkar, 2013). The conservation of the Aß molecular sequence throughout vertebrate evolution implies that it must confer a selective advantage for species survival. This notion is further supported by evidence that depletion of endogenous Aß has adverse consequences in a variety of species and animal models (summarized in Table 1). Although this concept runs counter to research that has focused on Aß's neurotoxic potential in AD, enough evidence has accumulated to suggest that Aß serves a beneficial role in human physiology, where it may contribute to:

- **Antimicrobial activity**: Aß has antibacterial, antifungal, and antiviral properties that are effective against at least eleven species of microbes.
- **Tumor suppression**: Aß may intercept oncogenic viruses and suppress tumor growth.
- **Sealing leaks in the blood-brain barrier (BBB):** Aß binds blood-borne solutes together to form a plug that prevents the spread of neuroactive and toxic components into the brain.
- **Promoting recovery from brain injury**: The presence of Aß results in better outcomes in animal models of controlled cortical impact, spinal cord injury, hypoxia, and autoimmune disease.
- **Regulating synaptic function**: Aß regulates the responsiveness of glutamatergic and cholinergic synapses in the hippocampus, thereby contributing to memory consolidation.

**Table 1:** Adverse consequences of endogenous Aß depletion.

**Experiment**	**Model (Strain)**	**Results**	**References**
BACE1 knockout	Mice (C57BL/6)	Worse motor performance following controlled cortical impact	Mannix et al., 2011a
BACE1 knockout or γ-secretase inhibition	Mice (C57BL/6)	More white matter damage and impaired locomotor recovery following spinal cord injury	Pajoohesh-Ganji et al., 2014
APP or BACE1 knockout	Mice (C57BL/6)	No compensatory increase in blood flow after cerebral ischemia, resulting in increased acute mortality	Koike et al., 2012
APP knockout	Mice (C57BL/6)	Worse progression of experimental autoimmune encephalomyelitis	Grant et al., 2012
Aß immunodepletion, blocking of Aß binding, or APP knockdown	Mice (CD-1 or C57BL/6)	Reduced hippocampal LTP and PTP, impaired spatial and contextual fear memory; rescued by treatment with human Aß_42_	Morley et al., 2010; Puzzo et al., 2011
BACE1 knockout	Mice (C57BL/6)	Spontaneous epileptic seizures, impaired spatial memory	Hu et al., 2010
BACE1 and BACE2 double knockout	Mice (mixed 129S5 and 129P2)	Increased mortality, reduced weight, hyperactive behavior	Dominguez et al., 2005
Aß immunodepletion	Rats (Long-Evans)	Impaired short- and long-term memory retention, rescued by treatment with human Aß_42_	Garcia-Osta and Alberini, 2009
BACE1 or γ-secretase inhibition or Aß immunodepletion	Rat (Wistar) cortical or cerebellar granule neurons, human SH-SY5Y cells	Reduced cell viability, rescued by incubation with human Aß_40_	Plant et al., 2003
Aß immunodepletion	Mouse lemur primates	Microhemorrhages, iron accumulation in the choroid plexus	Joseph-Mathurin et al., 2013
Anti-APP morpholino or ß-secretase inhibition	Zebrafish	Cerebrovascular defects, rescued by treatment with human Aß_40_	Luna et al., 2013

*APP, Aß precursor protein; BACE1, ß-site APP cleaving enzyme 1; PTP, post-tetanic potentiation; LTP, long-term potentiation*.

Such beneficial properties may explain the persistence of Aß throughout the vertebrate series. The following sections consider the evidence that supports each of these putative functions.

## Aß has antimicrobial properties

Among the first physiological functions of Aß to be proposed was the “Bioflocculant hypothesis” (Bishop and Robinson, 2002; Robinson and Bishop, 2002), where we noted that the widespread occurrence of Aß in healthy individuals suggests that Aß plays a natural physiological role, one that is most probably protective. We suggested that “*Aß may have a broader role as a general chelator and flocculant of potentially toxic agents that are dissolved in the extracellular fluid. In addition to metal ions, this would include bacteria and viruses, proteins, and neuroactive molecules that have been inadvertently released into the extracellular fluid.”* Once bound and taken out of solution, we envisaged that these pathogens could be phagocytosed and cleared by microglia and macrophages. In our review of the recent evidence for Aß's role as an antimicrobial peptide (AMP), a class of innate immune molecules with broad-spectrum antimicrobial properties, we noted that Aß not only binds and intercepts microbial pathogens, as suggested by the Bioflocculant hypothesis, but also possesses microbicidal activity that enables it to directly kill bacteria and viruses (Gosztyla et al., 2018).

The notion that Aß is an AMP is consistent with reports that several other amyloid peptides have antimicrobial properties, including serum amyloid A, microcin E492, temporins, and protegrin-1 (for reviews see Bishop and Robinson, 2004a; Kagan et al., 2012). Their antimicrobial activity may be partly due to the capacity of these peptides to form fibrils that insert into cell membranes and create pores that permit the unregulated passage of solutes into and out of microbes, leading to the death of these cells (Kagan et al., 2012). Similarly, Aß may capture and perforate microbes with its hairpin loop, while aggregates of Aß may immobilize microbes, akin to neutrophil extracellular traps, and the destruction of microbes may be accelerated by increased oxidation in the presence of iron from ferritin-rich cells like microglia (Batton et al., 1997; Robinson et al., 2003; Bishop and Robinson, 2004b; Wang et al., 2012).

The antimicrobial activity of human Aß was confirmed by Soscia et al. who demonstrated that the addition of 25 μg/mL of synthetic Aß_42_ slowed the proliferation of seven different bacteria and one fungal species in culture as effectively or better than the innate defensin LL-37. Aß_42_ was found to be slightly more potent than Aß_40_ when delivered at the same concentrations. It may be argued that this antimicrobial effect is not representative of the *in vivo* situation, because the concentrations of Aß used by Soscia et al. exceeded the physiological range by 4–5 orders of magnitude. However, this limitation was addressed by demonstrating that homogenates of temporal cortex from AD brain are more effective at inhibiting the growth of *Candida albicans* cultures than homogenates from cognitively normal subjects. This inhibitive effect was neutralized by pre-incubating the homogenates with anti-Aß antisera, indicating that the antimicrobial activity was probably due to the higher Aß burden in the AD brains (Soscia et al., 2010). Additionally, if Aß responds to pathogens, it is likely that the concentration of Aß in the immediate vicinity would far exceed the concentration of Aß averaged across a given brain region. These findings were confirmed by Spitzer et al. who found that 25–50 μg/mL of Aß_42_ agglutinated and reduced the viability of four bacterial species and the yeast *C. albicans* (Spitzer et al., 2016). Direct evidence for Aß's antimicrobial activity *in vivo* was reported in a recent study by Kumar et al. Here, mice and nematodes that overexpressed human Aß demonstrated enhanced resistance to bacterial or yeast infections. Electron microscopy images revealed that Aß fibrils entrapped bacterial and yeast cells *in vitro* and *in vivo* (Kumar et al., 2016).

In addition to bactericidal and fungicidal activity, Aß has virucidal properties (reviewed by Bourgade et al., 2016a). Lukiw et al. demonstrated that high concentrations of Aß_42_ inhibit the infection of human neuron-glia co-cultures by herpes simplex virus 1 (HSV1) as effectively as the antiviral agent acyclovir (Lukiw et al., 2010). An *in vitro* study by Bourgade et al. solidified this finding by demonstrating that Aß_42_ and Aß_40_ prevent HSV1 infection as effectively as LL-37, by binding to the virus and preventing its uptake into cells (Bourgade et al., 2015). This team further demonstrated that human H4 neuroglioma cells produce Aß upon exposure to HSV1 and that transfer of cell media containing Aß to naive H4 cells prevented HSV1 infection (Bourgade et al., 2016b). Aß was ineffective against the non-enveloped human adenovirus, leading Bourgade et al. to conclude that the antiviral activity of Aß is associated with a capacity to interact with viral coat proteins.

This conclusion is consistent with the findings of White et al. who showed that Aß_42_, and to a lesser extent Aß_40_, are effective at preventing infection of cultured cells by the pandemic strains H1N1 and H3N2 of the influenza virus (an enveloped virus) (White et al., 2014). The primary antiviral mechanism involves Aß binding viral particles into extracellular aggregates that were then precipitated from the supernatant and unable to infect cultured fibroblasts. Furthermore, pre-incubation of the virus with soluble Aß stimulated the subsequent uptake of virus by phagocytes but the virus did not replicate within these cells, indicating that the aggregated viral particles had been neutralized by the Aß. The strength of these effects increased as a function of Aß concentration. It appears that the antimicrobial property of Aß is largely due to its capacities to permeablize cells and to bind and aggregate pathogens. A recent study by the same group found that the Aß_42_ C-terminal amino acids 41 and 42 are critical for this function, as fragments lacking these amino acids show an impaired ability to flocculate and promote neutrophil uptake of viruses and bacteria (White et al., 2018).

Indirect evidence from animal studies has shown that the production of Aß waxes and wanes in response to immune challenge and healthy resolution. For example, Aß deposits have been noted in wild-type mice infected with *Chlamydia pneumoniae* (Little et al., 2004, 2014; Boelen et al., 2007), HSV1 (Wozniak et al., 2007), pseudorabies virus (Tanaka and Nagashima, 2017), or *Toxoplasma gondii* (Torres et al., 2018), while transgenic-AD mice infected with *Porphyromonas gingivalis* showed increased Aß deposition (Ishida et al., 2017). Notably, Aß returned to normal levels after the *C. pneumoniae* infection had resolved. In addition, wild-type mice infected with persistent cerebral toxocariasis acquire insoluble Aß deposits in the hippocampus at concentrations 10–20-fold higher than in uninfected mice (Chou et al., 2017). The presence of Aß deposits in non-transgenic mice is significant because it may provide insight into the presence of Aß in the 95% or more AD patients who have non-hereditary “sporadic” AD. This interpretation is further supported by evidence from human studies. CSF levels of soluble APP or Aß decrease during CNS infection (Sjögren et al., 2001; Gisslén et al., 2009; Jesse et al., 2010; Mattsson et al., 2010; Angel et al., 2012; Krut et al., 2013), indicating that APP and Aß_42_ are sequestered within the brain during this time. After resolution of Lyme neuroborreliosis and bacterial meningitis, CSF levels of APP, or Aß_42_ return to normal (Sjögren et al., 2001; Angel et al., 2012).

**Figure 1:**
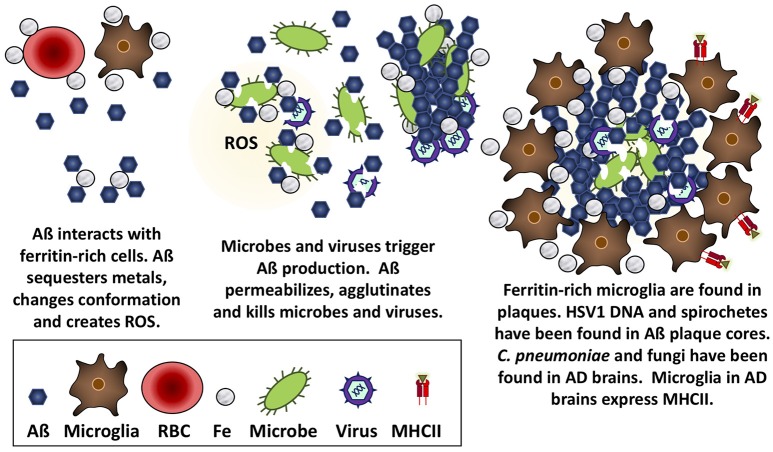
Aß is antimicrobial against bacteria, fungi, and viruses. Aß has the mechanical properties to trap microbes, insert into and permeabilize their membranes, and create a toxic oxidative response that is likely accelerated in the presence of iron obtained from nearby ferritin-rich cells.

Several researchers have championed the “Pathogen hypothesis” of AD (for review see Robinson et al., 2004b; Itzhaki et al., 2016), which postulates that AD may be caused by a cerebral infection of HSV1 (Ball, 1982; Ball et al., 2013; Itzhaki, 2014), *Borrelia* (Miklossy, 2015), or *C. pneumoniae* (Balin et al., 2008). While a discussion of that literature is beyond the scope of the present review, it is pertinent to note a variety of microbial species have been observed in Aß plaques or AD brains. Specifically, HSV1 DNA (Wozniak et al., 2009), and *Borrelia* antigen and DNA (Miklossy, 2016) have been found in plaque cores, both *Borrelia* and *C. pneumoniae* have been cultured from AD brain tissue (Balin et al., 1998; Gérard et al., 2006; Dreses-Werringloer et al., 2009), and extracellular and intracellular *C. pneumoniae* and various intraneuronal fungal infections have been reported in AD brain tissue (Hammond et al., 2010; Alonso et al., 2014, 2015; Pisa et al., 2015a,b). These observations can be viewed within the context of work by Michael D'Andrea and others, which have demonstrated that intracellular accumulations of Aß can burst out following cell death to produce extracellular dense-core plaques (reviewed by D'Andrea, 2014, 2016). A viral infection could contribute to intraneuronal deposition of Aß, resulting in cell lysis, and the release of dense-core plaques containing viral DNA into the extracellular space. In contrast, extracellular Aß accumulation could be attributed to the interception of bacterial or fungal pathogens. Taken together, these data suggest that Aß responds to and limits various types of infections in cells, animals, and humans (Figure 1).

Since Aß acts as an AMP, depletion of Aß would be expected to increase infection rates or infection severity. Indeed, clinical trials that have targeted Aß in AD patients have provided support for this notion (Table 2). For instance, approximately 6% of trial participants that received AN-1792, an active anti-Aß immunization, developed meningoencephalitis (Orgogozo et al., 2003; Robinson et al., 2004a; Gilman et al., 2005; Patton et al., 2006). Increased infection rates, including orolabial herpes relapse, and upper respiratory infections, have been reported in clinical trials of ß- or γ-secretase inhibitors [Green et al., 2009; Doody et al., 2013; AlzForum (n.d.-d, h, m)] and the Aß-binding compound ELND005 (Salloway et al., 2011; Alonso et al., 2014). A recent meta-analysis of ten clinical trials concluded that γ-secretase inhibitors are associated with an increased risk of infections (Penninkilampi et al., 2016). While these observations are not direct evidence of an antimicrobial role for Aß, they are certainly consistent with this possibility and indicate that future clinical trials should be alert to the potential for such outcomes.

**Table 2:** Adverse events in Aß-targeted clinical trials.

**Drug (Mechanism)**	**Aß endpoints**	**Relevant adverse effects**	**References**
Aducanumab (Anti-Aß immunization, Passive)	↓ Aß plaques	ARIA ↑ Urinary tract infection ↑ Upper respiratory tract infection	AlzForum (n.d.-a); Ferrero et al., 2016; Sevigny et al., 2016
Affitope AD02 (Anti-Aß immunization, Active)	↓ Aß plaques	↑ Atrophy and worsened cognition compared to unexpectedly beneficial placebo	AlzForum (n.d.-b); Schneeberger et al., 2009; AlzForum, 2014b
Alzhemed (Aß-binding)	↓ CSF Aß	↑ Hippocampal atrophy ↑ GI reactions	Aisen et al., 2006, 2007; Gauthier et al., 2009; Saumier et al., 2009
AN-1792 (Anti-Aß immunization, Active)	↓ Focal plaques NE total Aß NE/↑ vascular Aß ↑ Soluble Aß in gray matter ↑↑↑ Aß in white matter	↑ Atrophy transiently NE/↑ Atrophy on follow-up 6% meningoencephalitis 8 deaths with “virtually complete plaque removal”	AlzForum (n.d.-c) Nicoll et al., 2003; Ferrer et al., 2004; Fox et al., 2005; Masliah et al., 2005; Patton et al., 2006; Holmes et al., 2008; Kokjohn and Roher, 2009; Vellas et al., 2009; Boche et al., 2010
Avagacestat (γ-secretase inhibitor)	↓ Aß slightly in CSF	NE/worsened cognition ↑ Atrophy ↑ ARIA ↑↑ Skin cancer Skin/GI reactions	Coric et al., 2012, 2015
Bapineuzumab (Anti-Aß immunization, Passive)	↓ Fibrillar Aß	ARIA (~33% in APOE4/4, 7% APOE4/X, 4% non-APOE4), ↑ Seizures ↑ Paranoia ↑ Skin/GI/cardiovascular reactions	Salloway et al., 2009; Black et al., 2010; Rinne et al., 2010; AlzForum, 2012a,b; AlzForum (n.d.-e); Pfizer, 2008, 2013; AlzForum, 2013; Hu et al., 2015; Ketter et al., 2017
BI1181181 (BACE-inhibitor)	↓ CSF Aß ~80%	↑ Skin reactions	AlzForum (n.d.-f)
CAD106 (Anti-Aß immunization, Active)	↓ Brain Aß NE CSF Aß ↑ Plasma Aß 2-5X	↑ ARIA in strong responders ~8% Attrition for safety concerns ↑ Acute psychosis ↑ Skin/cardiovascular reactions	AlzForum (n.d.-g); AlzForum, 2014a
E2609 (BACE-inhibitor)	↓ Plasma Aß ↓ CSF Aß ~80%	↑ Infections ↑ Relapse of orolabial herpes	AlzForum (n.d.-h)
ELND005 (Aß-binding)	↓ CSF Aß	↑ Ventricular volume ↑ Infections 9 deaths at high doses	AlzForum (n.d.-i); Transition Therapeutics Ireland Limited, 2007, 2009; AlzForum, 2009; Salloway et al., 2011; Ma et al., 2012
Gantenerumab (Anti-Aß immunization, Passive)	↓ Aß ~11%	↑ ARIA (temporary and in areas with the most Aß reduction) “Futility”	AlzForum (n.d.-j);Ostrowitzki et al., 2012, 2017; Roche, 2014
LY2886721 (BACE-inhibitor)	↓ BACE activity 50–75% ↓ Aß_42_ ~72%,	↑ Abnormal liver biochemistry	AlzForum (n.d.-k)
RG7129 (BACE-inhibitor)		↑ Liver toxicity	AlzForum (n.d.-l)
Semagacestat (γ-secretase inhibitor)	NE/↓ CSF Aß ↓ Plasma Aß_40_ 38–72%	NE/worsened cognition ↑ Infections ↑ Skin cancer, skin reactions	AlzForum (n.d.-m); Siemers et al., 2006, 2007; Eli Lilly Company, 2008; Fleisher et al., 2008; Bateman et al., 2009; Doody et al., 2013

*Documented negative outcomes from clinical trials that have targeted Aß. Not all results are reported. Only drugs with reported effects or side-effects outside of Aß modulation are included on this table. ARIA, Amyloid-related imaging abnormalities; APOE, apolipoprotein; NE, no effect*.

## Aß may protect against some forms of cancer

There is an impressive inverse relationship between AD and cancer. One interesting example is the naked mole rate, a notoriously cancer-resistant rodent that accumulates Aß at levels similar to AD-mice bearing at least three human transgenes without developing memory impairment (Edrey et al., 2013; Deweerdt, 2014). Multiple studies have demonstrated that cognitively normal elderly patients who are diagnosed with cancer are less likely to subsequently develop AD, whereas patients who have been diagnosed with probable AD are half as likely to have had cancer or to develop cancer compared to age-matched, cognitively-normal peers (Driver et al., 2012; White et al., 2013; Catalá-López et al., 2014; Ma et al., 2014; Shi et al., 2015; Yarchoan et al., 2017). While some reports have suggested that this relationship may be due to ascertainment bias (Freedman et al., 2016; Bowles et al., 2017; Hanson et al., 2017), a recent examination of nearly 3.5 million veterans found that the risk of several types of cancer is lower in AD patients, even after accounting for bias (Frain et al., 2017).

AD patients have significantly lower incidences of non-melanoma skin cancer, head and neck cancer, colorectal cancer, lung cancer, breast cancer, bladder cancer, and hematologic malignancies (reviewed by Shi et al., 2015). A recent analysis of 4,357 subjects observed a reduced risk of AD following a diagnosis with incident cancer, though no difference in AD risk was found for prevalent cancers. However, when the analysis was restricted to late-stage prevalent cancers, diagnosis was associated with a 50% reduction in AD or dementia risk (Bowles et al., 2017). The fact that patients with vascular dementia have incidences of cancer that are comparable to those in the general elderly population (Roe et al., 2010) implies that there is something specific about AD that confers protection against cancer. This conclusion is supported by a meta-analysis of over 50 clinical studies involving more than half a million participants, in which it was found that AD is associated with a greatly reduced risk of cancer (Catalá-López et al., 2014).

While the basis of the protection against cancer is unknown, the outcomes of the AD clinical trials do not support a direct role for Aß in the repression of cancer. While some of the trials reported increased rates of cancer, such trials involved γ-secretase inhibitors, rather than inhibitors of BACE1 or immunotherapy against Aß (Table 2). For instance, clinical trials of the γ-secretase inhibitor Avagacestat were halted early, in part because seven patients developed squamous-cell or basal-cell carcinomas of the skin, compared to none in the placebo group (Coric et al., 2012, 2015). Similarly, a phase III trial of the γ-secretase inhibitor Semagacestat was discontinued after 5–6% of patients developed squamous-cell carcinomas of the skin and 15–16% developed “neoplasms,” compared to the placebo group who had rates of 1 and 5%, respectively (Doody et al., 2013). A meta-analysis found that γ-secretase inhibitors are associated with a nearly five-fold increase in skin cancer risk (Penninkilampi et al., 2016). It should be noted that increased rates of cancer have not been reported for clinical trials that have specifically targeted Aß, so it is likely that the adverse effects were related to functions of γ-secretase that are separate from its cleavage of Aß, such as a loss of the APP cleavage product, Notch (Roperch et al., 1998; Paris et al., 2005).

Although the evidence from clinical trials does not support an active role for Aß in the suppression of cancer, it is possible that circulating Aß plays an indirect role by intercepting oncogenic viruses. Up to 18% of cancers are thought to be induced by oncogenic viruses (Parkin, 2006). For example, most non-melanoma skin cancers, including squamous-cell and basal cell carcinomas, contain human papillomavirus (Arroyo Mühr et al., 2015). It is notable that these forms of cancer are underrepresented in AD. The finding that the oncogenic virus Epstein-Barr stimulates the production of anti-Aß antibodies, raises the possibility that some viruses may benefit from the elimination of Aß (Xu and Gaskin, 1997). Since high titers of anti-Epstein-Barr virus antibodies in patients with mild cognitive impairment are predictive of future cognitive decline (Shim et al., 2017), it is tempting to speculate that AD may involve attempts by oncogenic viruses to neutralize the defenses provided by Aß, which are then countered by increased production of antibodies against the virus. Such counter-responses could account for the lower rate of some cancers in AD.

Experimental evidence indicates that Aß is capable of inhibiting tumor cell growth. For instance, the treatment of cultured cancer cell lines with conditioned media containing Aß significantly reduced the rate of proliferation of human glioblastoma, human breast adenocarcinoma, and mouse melanoma cells (Zhao et al., 2009). The extent of the reduction was associated with the concentration of Aß present in the medium and was not linked to the presence of APP. In mice, Aß suppresses tumor growth when injected directly into human glioblastoma and human lung adenocarcinoma xenografts (Paris et al., 2004). Similarly, Aß delivered into the peritoneal cavity reduces the growth of lung adenocarcinoma xenografts (Paris et al., 2004). In transgenic mouse lines that overexpress Aß, the rates of growth of implanted glioma tumor masses are suppressed by 40–50% at 8 months of age compared to tumor masses in wild-type mice (Paris et al., 2010).

Paris et al. demonstrated that high concentrations of Aß inhibit capillary growth both *in vivo* and *in vitro*, and when present at very high concentrations it causes capillaries to degenerate. They concluded that Aß may slow tumor growth by retarding neovascularization (Paris et al., 2004, 2010). An alternative possibility, suggested by the present authors, relates to the exceptionally high binding affinity of Aß for iron, copper, and zinc (Bishop and Robinson, 2002; Robinson and Bishop, 2002). By scavenging free metal ions, Aß may limit the availability of these essential micronutrients and slow the proliferation of tumor cells. Evidently there are several potential mechanisms that could account for the inverse relationship between AD and some forms of cancer, and until further research has been conducted, the basis of this relationship will remain a matter for speculation.

## Aß seals leaks in the blood-brain barrier

In contrast to cancer, the link between Aß and the integrity of the BBB is firmly established. Aß plaques in AD brain tissue contain many different blood proteins and peptides, including serum albumin, fibrinogen, thrombin, IgG, von Willebrand factor, collagen IV, and hemin (Cullen et al., 2006), which are normally foreign to the brain. Hemoglobin also binds to Aß in an iron-dependent manner and colocalizes with Aß plaques and vascular deposits in *post-mortem* AD brains (Oyama et al., 2000; Wu et al., 2004; Chuang et al., 2012). In 2002, one of us proposed that if the BBB becomes leaky, allowing pro-inflammatory and neuroactive compounds to enter the brain, soluble Aß will bind these compounds into an insoluble mass to prevent their spread through the neuropil (Bishop and Robinson, 2002). Other researchers have built on this idea. Stone demonstrated that practically all plaques in AD are closely associated with a capillary, thereby supporting a causal link between a leaky BBB and Aß deposition (Stone, 2008), while Atwood et al. (2003) proposed that Aß may serve as a vascular “scab” that seals breaches of the BBB.

**Figure 2:**
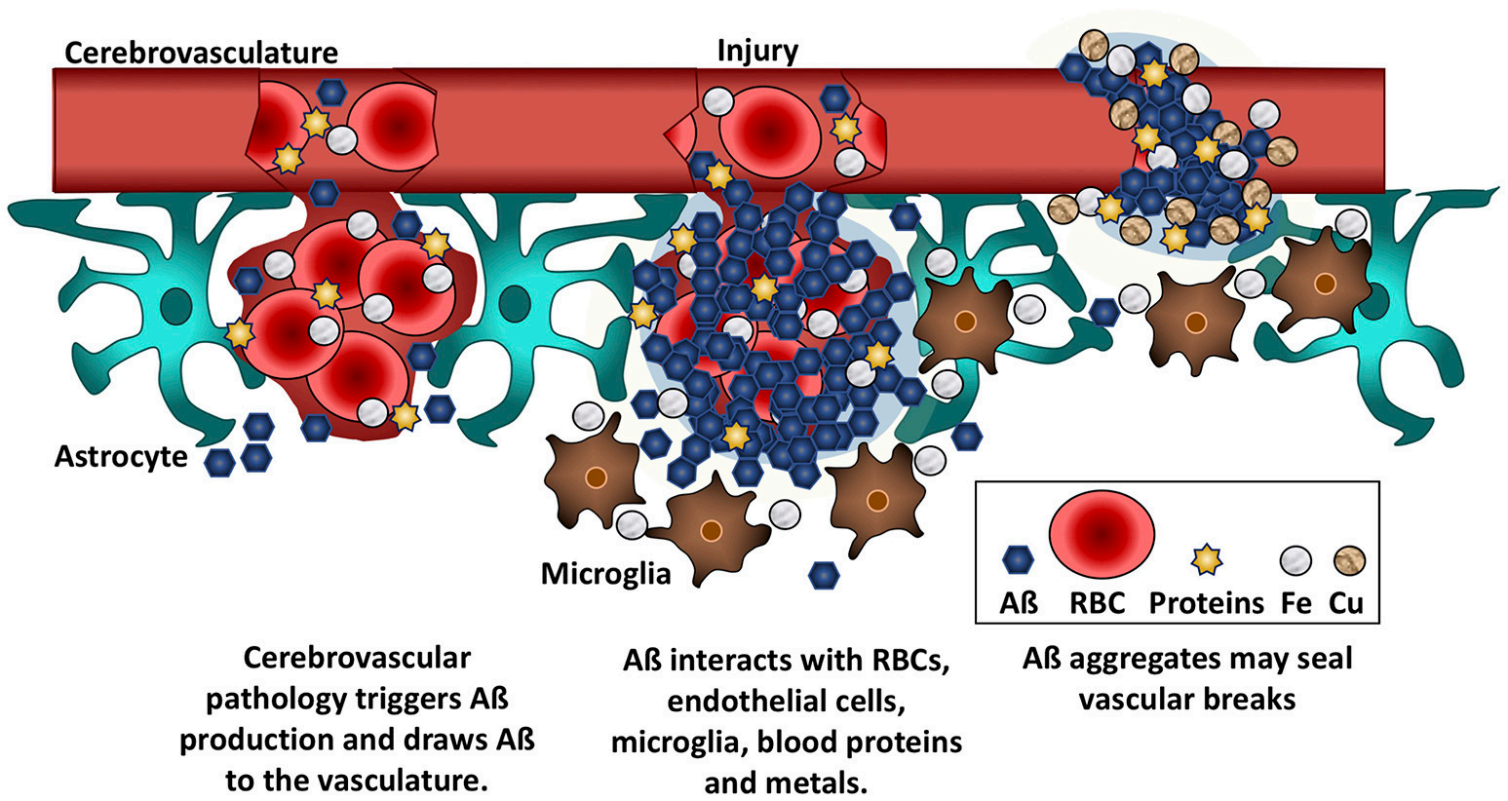
Aß seals leaky vessels. Traumatic brain injury and cerebrovascular insults stimulate Aß production and draw Aß to the vasculature. Aß binds to red blood cells (RBCs), blood proteins, and to iron (Fe) and copper (Cu) ions. These interactions cause Aß to aggregate at the site of hemorrhage or breaches of the BBB. Aß anchors into cell membranes and increases adherence between RBCs and vascular endothelial cells, helping to seal leaks in the vasculature.

Aß may slow bleeding with filamentous aggregates that pull the walls of the capillary endothelial cells back together (Atwood et al., 2003). In fact, incorporation of Aß into the surface of either red blood cells or endothelial cells increases the probability that the cells will adhere to the microvasculature (Ravi et al., 2004; Figure 2). Viewed from this perspective, the heavier plaque burden in AD may be because the BBB is more porous than in non-demented elders. A recent imaging study reported that the BBB becomes more permeable in the human hippocampus with age, and that permeability is more pronounced in individuals with mild cognitive impairment than in age-matched controls or those with multiple sclerosis (Montagne et al., 2015). In support of this hypothesis, cortical superficial siderosis is seven times more common in AD than in age-matched controls (Dubessy et al., 2012; Wollenweber et al., 2014); this condition is due to the accumulation of iron from extravasated hemoglobin, and its presence is indicative of a history of micro-hemorrhages (Charidimou et al., 2015). It follows that removal of Aß in AD is likely to lead to increased BBB permeability and an increase in micro-hemorrhages and subsequent brain edema.

Clinical trials targeting Aß have provided dramatic confirmation of this hypothesis. The most common adverse side-effect of these trials has been MRI evidence of brain edema and/or micro-hemorrhages, sometimes accompanied by increased confusion and disorientation. This pattern of pathological change, so characteristic of amyloid depletion, has been termed “Amyloid-Related Imaging Abnormalities” (ARIA). The prevalence of ARIA has been very high in clinical trials of passive immunotherapies (e.g., Bapineuzumab, Solenezumab, Aducanumab, and Gantenerumab) (reviewed by Lannfelt et al., 2014; DiFrancesco et al., 2015). In the clinical trials with Bapineuzumab for instance, the incidence of ARIA with edema increased from 7.1% of patients on the lowest dose of the drug to 30.8% of patients on the highest dose; 47.2% of these patients also exhibited evidence of micro-hemorrhages, while a further 4% had micro-hemorrhages without evidence of increased edema (Sperling et al., 2012). In the Aducanumab trials, the incidence of ARIA was even higher, at 47–55% of participants in the highest dosage group (AlzForum (n.d.-a); DiFrancesco et al., 2015; Sevigny et al., 2016). A meta-analysis of fourteen clinical trials found that anti-Aß immunotherapies are associated with a nearly five-fold increase in ARIA (Penninkilampi et al., 2017). Unlike trials targeting secretases, these immunotherapies targeted Aß without altering APP processing, suggesting that ARIA is directly related to the loss of Aß. ARIA resulting from the targeting of Aß is more likely to occur in carriers of the apolipoprotein ε4 (APOE ε4) allele AlzForum (n.d.-e), and imaging confirms that the edema occurs at focal sites that exhibit the greatest reductions in Aß (Ostrowitzki et al., 2012). The edema results from the entry of blood solutes into the neuropil, followed by an influx of water into the brain along the osmotic gradient.

Although ARIA was not observed in the early experiments with transgenic mice, subsequent experiments have confirmed that the link exists. For instance, anti-Aß immunotherapy of PDAPP mice, which overexpress APP, leads to an increase in BBB permeability in a subset of mice that is accompanied by cerebral microbleeds, siderosis, and localized edema, which are the hallmarks of ARIA (Blockx et al., 2016). Similarly, aged mouse lemur primates, which exhibit an age-associated accumulation of endogenous Aß with a peptide sequence that is similar to that in humans, also develop ARIA following anti-Aß immunotherapy (Joseph-Mathurin et al., 2013).

Several lines of experimental evidence from mice indicate that BBB breakdown leads to increased Aß deposition. Mice that overexpress endothelin-1, resulting in a weakened BBB, show increased astrocytic secretion of Aß following an ischemic stroke (Hung et al., 2015). Micro-hemorrhages created with Rose Bengal dye in transgenic-AD mice drove transient increases in Aß plaques in infarcted and adjacent areas (Garcia-Alloza et al., 2011). Similarly, micro-hemorrhages created with diet-induced hyperhomocysteinemia in transgenic-AD mice shifted the distribution of Aß deposits from the parenchyma to the vasculature (Sudduth et al., 2014). Furthermore, hemorrhages induced by needle stick lesions in wild-type rats led to a transient up-regulation of APP, Aß, and phosphorylated tau near the lesion site and a longer-lasting deposition of Aß along the needle tract (Purushothuman et al., 2013). Mice that have been chronically subjected to high blood pressure develop Aß deposits around their cerebral blood vessels and display learning impairments (Carnevale et al., 2016).

Collectively, the preceding observations provide powerful support for the view that Aß seals leaks in the BBB, a role that probably becomes increasingly important as the aging BBB gradually loses its integrity. Viewed from this perspective, comorbid conditions that are likely to enhance the permeability of the BBB, such as diabetes and vascular hypertension, should be correlated with a heavier plaque burden. Indeed, a diabetic phenotype does increase the expression of Aß in AD-Tg mice (Ho et al., 2004), is sufficient to do the same in wild-type rabbits (Bitel et al., 2012), and is associated with greater plaque pathology in the subset of diabetic AD patients with an APOE ε4 genotype (Malek-Ahmadi et al., 2013). Similarly, vascular hypertension is associated with higher Aß burdens in wild-type animal models (Gentile et al., 2009; Schreiber et al., 2014; reviewed by Bueche et al., 2014), increased Aß aggregates in the human placenta (Kalkunte et al., 2013; Buhimschi et al., 2014), and increased Aß deposits in the AD brain (Ashby et al., 2016).

## Aß may improve recovery from brain injury

The presence of Aß plaques in the hippocampus and cerebral cortex has become synonymous with AD, to the point where cognitively normal persons with significant Aß burdens are assumed to have incipient AD. However, as was seen in the preceding sections, the presence of Aß deposits does not necessarily indicate AD; they may indicate sites where pathogens have been intercepted and neutralized or where a leaky BBB has been repaired. Another well-documented role of Aß is in assisting the brain to recover from traumatic and ischemic injuries.

In humans, a traumatic brain injury (TBI) elevates APP levels in the brain within 2 h (McKenzie et al., 1996) and Aß plaques become evident within 4 h (Roberts et al., 1994; Graham et al., 1995; Johnson et al., 2010). Two microdialysis studies of brain extracellular fluid from TBI patients found that those with the higher titers of Aß experienced better outcomes (Brody et al., 2008; Magnoni et al., 2012). Aß plaques form routinely after a head injury, even in children as young as 10 years, who presumably would not otherwise have plaques (Roberts et al., 1994; Graham et al., 1995), and they are more likely to form in APOE ε4 carriers (Nicoll et al., 1995; Zunarelli et al., 1996; Mauri et al., 2006; Abu Hamdeh et al., 2017). A PET study of TBI patients found that while Aß deposits colocalized with areas of white matter damage, the Aß burden was not significantly correlated with the extent of neuropsychological impairment (Scott et al., 2016). Though Aß accumulation occurs immediately after a traumatic injury and can persist in damaged axons for years (Johnson et al., 2012; Scott et al., 2016; Bagnato et al., 2017), the brains of long-term survivors of head injury do not have greater plaque numbers or increased APP expression when compared to age- and APOE-matched controls (Macfarlane et al., 1999; Chen et al., 2009). This suggests that Aß accumulates transiently in response to injury.

Evidence from animal models also shows that Aß expression responds rapidly to injury, resolves over time, and may be necessary for a good recovery. A recent meta-analysis of 19 animal studies reported that Aß expression consistently increases within 24 h of TBI, including in models that lack AD-related transgenes (Bird et al., 2016). For example, controlled cortical impact leads to an upregulation of APP and BACE1 expression in wild-type rats (Blasko et al., 2004; Acosta et al., 2017) and accelerates Aß deposition in transgenic-AD mice (Tajiri et al., 2013; Washington et al., 2014; Shishido et al., 2016). Controlled cortical impact increases the expression of Aß_40_ and Aß_42_ in transgenic-AD mice within one day, with levels returning to baseline within one week (Washington et al., 2014). During this early period, post-injury macrophage activation is suppressed (Kokiko-Cochran et al., 2016), which may provide injured neurons with time to repair and recover, instead of being phagocytosed.

It is notable that controlled cortical impact results in worse motor performance in mice that are deficient in BACE1 compared to wild-type mice (Mannix et al., 2011a), and when the mice are treated with intra-ventricular injections of Aß_40_ after the injury, motor performance improves in the knock-out mice but worsens in the wild-type mice (Mannix et al., 2013). These results suggest that the level of Aß production in wild-type mice after controlled cortical impact was tuned and appropriate. Recovery after controlled cortical impact is also modulated by age and APOE genotype; in immature and adult mice expressing human APOE ε4, only adults showed worse spatial memory performance compared to wild-type, as well as high Aß_40_ levels 1 month after injury (Mannix et al., 2011b). Similarly, Aß expression increases during the first 3 days after spinal cord injury, and if Aß production is prevented by BACE1 knock-out or by treatment with a γ-secretase inhibitor, the mice develop more white matter damage, and display impaired recovery from locomotor deficits (Pajoohesh-Ganji et al., 2014).

Steinman et al. noted that Aß_42_ is prominent in cerebral lesions and in the damaged axons of patients with multiple sclerosis, and postulated that this might represent a protective response to injury (Ferguson et al., 1997; Trapp et al., 1998; Han et al., 2008). Steinman's group delivered intraperitoneal injections of Aß_40_ or Aß_42_ into four different animal models of multiple sclerosis. Stunningly, they found that the treatment attenuated motor paralysis, reduced the extent of demyelinated lesions, suppressed lymphocyte activation, and lowered pro-inflammatory cytokine expression in blood. In contrast, APP knockout mice fared much worse than wild-type mice. It is important to note that protection in this model was associated with a hexameric form of Aß that reduces T-cell activation (Grant et al., 2012).

Another example of the protection that Aß affords from brain injury comes from experimental models of stroke. In wild-type mice, occlusion of the common carotid artery drives a compensatory increase in blood flow in the cerebral arteries, but this compensatory increase is attenuated in APP knock-out mice (Koike et al., 2012). This reduction of compensatory blood flow is lethal, and consequently APP knock-out mice die shortly after bilateral occlusion of the common carotid artery, whereas wild-type mice survive; since BACE1 knockout mice suffer the same fate, this loss of viability is likely due to the absence of Aß rather than other products of APP cleavage (Koike et al., 2012). Further evidence that Aß is protective during a stroke comes from a rat model of middle cerebral artery occlusion, in which the mean infarct volume is significantly reduced in transgenic-AD rats compared to wild-type rats (Clarke et al., 2007).

The preceding observations show that the presence of Aß improves outcomes after injury to the central nervous system. Consequently, pre-emptive anti-Aß treatments for AD are likely to increase the probability that the treated individuals will display poorer prognoses if they have the misfortune of sustaining a TBI (e.g., from a fall) or a stroke.

## Aß may regulate activity at hippocampal synapses

A growing body of evidence demonstrates that soluble Aß is necessary for synaptic plasticity and memory (reviewed by Puzzo and Arancio, 2013; Morley and Farr, 2014). During periods of neuronal activity, APP is transported anterogradely to synapses, where Aß is cleaved and released along with neurotransmitter into the synaptic cleft (Kamenetz et al., 2003; Cirrito et al., 2005; Tampellini et al., 2009). Aß then acts on presynaptic neurons to increase the probability of further neurotransmitter release (Fedele et al., 2015; Puzzo et al., 2015). Depletion of endogenous Aß in rodents greatly reduces LTP and short- and long-term memory; this can be rescued by the addition of human Aß_42_ (Garcia-Osta and Alberini, 2009; Morley et al., 2010; Puzzo et al., 2011). Additionally, rodents treated with picomolar concentrations of human Aß_42_ show enhanced memory compared to a scrambled control peptide or vehicle (Garcia-Osta and Alberini, 2009; Morley et al., 2010). The duration of Aß exposure seems to be important for this process. Mouse hippocampal neurons exposed to physiological concentrations of oligomeric Aß_42_ show enhanced plasticity within minutes, but reduced plasticity with prolonged exposure (Koppensteiner et al., 2016). This was confirmed *in vivo*, as brief hippocampal infusions of Aß_42_ enhanced contextual memory in mice, while longer infusions impaired memory (Koppensteiner et al., 2016).

Aß may enhance long-term potentiation (LTP) by increasing the amount of acetylcholine released into the synaptic cleft and increasing the probability that the postsynaptic neuron will depolarize. Mice injected with low concentrations of Aß into the hippocampus showed enhanced memory retention in two memory tasks and increased acetylecholine production in the hippocampus (Morley et al., 2010). Picomolar concentrations of Aß directly activate α7-nicotinic acetylcholine receptors, whereas nanomolar concentrations of Aß block and inactivate the receptors. Similarly, picomolar concentrations of Aß_42_ enhance LTP, and memory consolidation in mice, while nanomolar concentrations impair memory (Fedele et al., 2015; Puzzo et al., 2015; Ricciarelli and Fedele, 2017). Furthermore, Aß-mediated enhancement of memory is ineffective in the absence of α7-nicotinic acetylcholine receptors (Fedele et al., 2015; Puzzo et al., 2015; Ricciarelli and Fedele, 2017).

In addition to interacting with acetylcholine signaling, Aß can also stimulate glutamatergic receptors. Nanomolar concentrations of Aß facilitate NMDA (N-methyl-D-aspartate) receptor-mediated LTP, while picomolar concentrations enhance contextual fear memories. When Aß is present at high picomolar concentrations (which are pathological), it can disrupt the clearance of glutamate from the extracellular space by astrocytes, leading to a build-up of extracellular glutamate that then causes aberrant activation of NMDA receptors and eventual synaptic dysfunction (Tu et al., 2014).

Some clinical trials that have depleted patients' brains of Aß have reported increased rates of adverse events that might be attributable to synaptic dysfunction. For instance, increased seizure activity was reported in clinical trials of the anti-Aß immunotherapy Bapineuzamab (AlzForum (n.d.-e)). In other clinical trials the removal of Aß has been accompanied by worse cognitive outcomes. For example, the γ-secretase inhibitor Semagacestat caused significantly lower scores on tests of cognitive status, functional status, and dementia in a dose-dependent manner, which did not resolve until 32 weeks after termination of drug dosing (Doody et al., 2013). Avagacestat, another γ-secretase inhibitor, was also associated with a decrease in performance on these same cognitive tests, though it did not reach statistical significance (Coric et al., 2012, 2015). In trials of active anti-Aß immunotherapies, Affitope AD02 counteracted the surprisingly beneficial effect of the placebo on cognitive function (AlzForum (n.d.-b); Schneeberger et al., 2009; AlzForum, 2014b), while CAD106 caused an increase in acute psychosis (AlzForum (n.d.-g); AlzForum, 2014a). The negative cognitive effects of anti-Aß immunotherapies support a direct role of Aß depletion in contributing to synaptic dysfunction in these trials.

While most animal studies have not reported adverse cognitive or behavioral effects after immunization with Aß, two anti-Aß immunotherapies (one was the rodent equivalent of Bapineuzumab) were recently tested in two transgenic mouse models of AD and all four conditions resulted in neuronal hyperactivity and dysfunction, independent of the effects of these antibodies on the clearance of Aß plaques (Busche et al., 2015). Notably, however, others have reported that anti-APP/Aß immunotherapy successfully reduced neuronal hyperexcitability and epileptiform discharges in triple transgenic-AD mice (Kazim et al., 2017). This may be explained by the use of older mice in the former study, compared to younger pre-plaque mice in the latter. Additionally, the inability of the antibody used in the latter study to differentiate between APP and Aß could also be a contributing factor to the discrepancy. Other studies have reported that the absence of APP and Aß, due to the knockout of APP or BACE1, increases spontaneous seizure activity and potentiates elicited seizures (Steinbach et al., 1998; Hu et al., 2010). Finally, the treatment of healthy wild-type mice with Aß immunotherapy or with antisense directed at APP significantly impaired their learning on a T-maze foot-shock avoidance task (Morley et al., 2010). Collectively, the evidence reviewed in this section provide strong evidence for Aß serving a physiological role in hippocampal LTP and memory retention.

## Conclusion

The research reviewed in the current paper reveals that the Aß peptide is involved in the protection and repair of the central nervous system. Aß regulates synaptic function and contributes to memory consolidation; it may also protect from some forms of cancer and aid recovery from TBI. There is solid evidence that pathogens or a breach in the BBB trigger soluble Aß to aggregate into insoluble deposits in order to intercept the pathogen or seal the leak. Some of the adverse outcomes associated with clinical trials can be understood from this perspective: a reduction in the capacity to intercept pathogens leads to a higher incidence of infections, while a loss of capacity to seal leaks in the BBB leads to increased numbers of micro-bleeds and brain edema (ARIA). This being the case, targeting the production or removal of Aß earlier in the course of AD is likely to be associated with the same adverse events, except that the longer duration between start of treatment and patient death will increase the likelihood of these adverse events occurring during the lifetime of the patient and may reduce the patients' capacity to recover.

More favorable outcomes might be achieved by treating the known triggers of Aß deposition before targeting Aß production. This would involve screening patients for potential causes of BBB leakage (such as diabetes or vascular hypertension) and/or for evidence of latent microbial infections, and then treating accordingly. Such treatments should slow the rate of Aß deposition, with corresponding benefits for cognition. Once these known sources of Aß deposition have been addressed, we would expect subsequent anti-Aß therapies to be associated with fewer instances of ARIA or brain infection. However, it remains possible that anti-Aß therapies will adversely affect learning and memory, recovery from TBI or the incidence of some forms of cancer.

## Author contributions

HB: Wrote the first draft of the manuscript; MG and SR: Substantially revised subsequent drafts. All authors have read and approved the final manuscript.

### Conflict of interest statement

The authors declare that the research was conducted in the absence of any commercial or financial relationships that could be construed as a potential conflict of interest.

## Abbreviations

Aß: Amyloid-ß
AD: Alzheimer's disease
APP: amyloid-ß precursor protein
AMP: antimicrobial protein
BACE1: β-site APP cleaving enzyme 1
HSV1: herpes simplex virus-1
APOE: apolipoprotein
CSF: cerebrospinal fluid
ARIA: amyloid-related imaging abnormalities
BBB: blood-brain barrier
TBI: traumatic brain injury
LTP: long-term potentiation.
